# Practical Considerations of Dissolved Oxygen Levels for Platelet Function under Hypoxia

**DOI:** 10.3390/ijms222413223

**Published:** 2021-12-08

**Authors:** Branden Kusanto, Andrew Gordon, Leigh Naylor-Adamson, Lloyd Atkinson, Charlie Coupland, Zoe Booth, Yusra Ahmed, Isabel M. Pires, Graeme J. Stasiuk, Roger Sturmey, Simon D. J. Calaminus, Mònica Arman

**Affiliations:** 1 Centre for Atherothrombosis and Metabolic Disease, Hull York Medical School, Faculty of Health Sciences, University of Hull, Hull HU6 7RX, UK; branden.kusanto@gmail.com (B.K.); Andrew.Gordon@hyms.ac.uk (A.G.); Leigh.Naylor-Adamson@hyms.ac.uk (L.N.-A.); Lloyd.Atkinson@hyms.ac.uk (L.A.); hycc29@hyms.ac.uk (C.C.); hyzb2@hyms.ac.uk (Z.B.); yusra.ahmed@hee.nhs.uk (Y.A.); Roger.Sturmey@hyms.ac.uk (R.S.); Simon.Calaminus@hyms.ac.uk (S.D.J.C.); 2 Department of Biomedical Sciences, Faculty of Health Sciences, University of Hull, Hull HU6 7RX, UK; i.pires@hull.ac.uk; 3 Department of Imaging Chemistry and Biology, School of Biomedical Engineering and Imaging, King’s College London, St. Thomas’ Hospital, London SE1 7EH, UK; graeme.stasiuk@kcl.ac.uk

**Keywords:** platelet, platelet rich plasma, whole blood, hypoxia, dissolved oxygen concentration, in vitro assay

## Abstract

Investigating human platelet function in low-oxygen environments is important in multiple settings, including hypobaric hypoxia (e.g., high altitude), sea level hypoxia-related disease, and thrombus stability. These studies often involve drawing blood from which platelets are isolated and analysed at atmospheric conditions or re-exposed to low oxygen levels in hypoxia chambers before testing. However, it remains unknown how the in vitro handling of the samples itself changes their dissolved oxygen concentration, which might affect platelet function and experimental results. Here, we prepared healthy donor platelet-rich plasma and washed platelet (WP) suspensions and exposed them to 2% oxygen. We found that the use of hypoxia pre-equilibrated tubes, higher platelet concentrations (>2 × 10^8^/mL versus 2 × 10^7^/mL), smaller volumes (600 µL versus 3 mL), and presence of plasma reduced the time for samples to reach 2% oxygen. Notably, oxygen levels decreased below 2% in most suspensions, but also in WP maintained at atmospheric 21% oxygen. Additionally, platelet spreading on fibrinogen was decreased when using hypoxic fibrinogen-coated culture plates regardless of the oxygen percentage (2% or 21%) in which platelet incubation took place. Thus, sample handling and experimental conditions should be carefully monitored in platelet-hypoxia studies as they might compromise results interpretation and comparison across studies.

## 1. Introduction

The importance of understanding the relationship between low oxygen concentration and platelet function in physiology and pathology has been highlighted in a growing number of publications. Exposure to hypobaric hypoxia (e.g., ascending to high altitude) has been associated with a thrombogenic phenotype [1,2,3,4] and can induce changes in platelet proteome and ADP signalling pathways [5,6,7]. At sea level, chronic hypoxic diseases such as obstructive sleep apnoea and chronic obstructive pulmonary disease have been reported to have hyperactive platelets in circulation and an increased risk of thrombosis [8,9,10,11,12,13]. Moreover, platelet activation can contribute to hypoxia-induced inflammation [14]. In the case of thrombotic and vascular diseases resulting in localised hypoxia (e.g., peripheral arterial disease), alterations in platelet agonist sensitivity and platelet phenotype have been reported [15], which might affect the success of antiplatelet therapy. These phenotypic alterations include changes in surface receptor expression, intracellular signalling and proteomic profiles, and are also observed in platelets from healthy donors exposed to low oxygen for a short period [15]. While some of these changes might be irreversible, a fast but reversible impairment of integrin αIIbβ3 activation has also been reported [16]. Deciphering platelet function and intracellular signalling at sub-atmospheric oxygen levels is also critical to understand thrombus stability, as platelets in the core of a thrombus might have restricted access to oxygen but still perform energy-consuming activities. A recent study reported an increase in hypoxia-inducible factor 2α (HIF-2α) expression upon platelet exposure to hypoxia, as well as in circulating platelets from individuals living at high altitude and patients with chronic obstructive pulmonary disease [17].

While the above findings emphasise the importance of understanding the relationship between low oxygen concentration and platelet function, most of these studies used different experimental approaches, which renders results comparison difficult. Much of our knowledge of human platelet function is derived from ex vivo experiments, where platelets are exposed to atmospheric oxygen levels that do not match the prevailing conditions they normally experience in vivo. Similarly, in the case of hypoxia-focused studies, one main research strategy involves obtaining blood samples from human volunteers (or other animals) exposed to hypobaric hypoxia or patients with hypoxia-related disease, which are then processed and analysed at atmospheric conditions (21% oxygen). Another strategy is to re-expose platelets isolated from healthy donors or patients to sub-atmospheric oxygen levels, which can vary across studies, in hypoxia chambers. The latter is important to study the direct effects of hypoxia on platelets, rather than indirect effects through the vascular system and megakaryocytes. In the case of in vitro (non-platelet) cell cultures, the influence of experimental manipulations on de-oxygenation and re-oxygenation has been well documented and can affect cell behaviour and assay outcome [18,19]. Even though platelets are anucleate, and platelet suspensions are often used within a few hours of sample preparation, it is also possible that the in vitro handling of the samples itself affects the oxygen concentration in the cell suspensions, which might in turn affect experimental results.

This study aimed to investigate how different platelet preparations used widely for platelet testing are affected by exposure to low oxygen levels, to help identify the extent to which data on the effect of hypoxia on human platelets might be influenced by experimental conditions. To this end, whole-blood, platelet-rich plasma (PRP) and washed platelets (WP) from healthy human volunteers were exposed to 2% oxygen in varying tubes, platelet concentrations and volumes; and the de-oxygenation and re-oxygenation (after re-exposure to sea level atmospheric conditions) of the cell suspensions were measured over time. Additionally, platelet spreading assays on fibrinogen were performed to analyse whether experimental conditions could affect platelet functional responses in a low-oxygen environment.

## 2. Results

### 2.1. Effect of Tubes on Oxygen Level Changes in Platelet Suspensions Exposed to 2% Oxygen

To determine whether platelet sample preparation could affect the de-oxygenation rate of platelet suspensions exposed to hypoxia, we first tested different tubes. WP (2.5 × 10^8^ platelets/mL) and undiluted PRP (4 to 8 × 10^8^ platelets/mL, donor-dependent concentration) were prepared, and dissolved oxygen concentrations were read by inserting an oxygen probe microsensor into the middle of the samples (Time “0”). Platelet suspensions were then introduced into a hypoxia chamber set at 2% oxygen and 37 °C. Immediately after entering the chamber, 600 µL of WP and PRP were aliquoted in 1.5 mL polypropylene microcentrifuge tubes prepared in three different ways: (1) tubes pre-incubated overnight (15 h or longer) in 2% oxygen with lid closed on one side during platelet incubation, (2) tubes pre-incubated overnight in 2% oxygen and covered with Parafilm instead of closing the lid, and (3) tubes kept at atmospheric conditions until time 0, when they were placed in the hypoxia chamber and kept partially closed during platelet incubation. Tubes with platelet samples were only fully opened when performing oxygen measurements, which only took a few seconds each.

**Figure 1:**
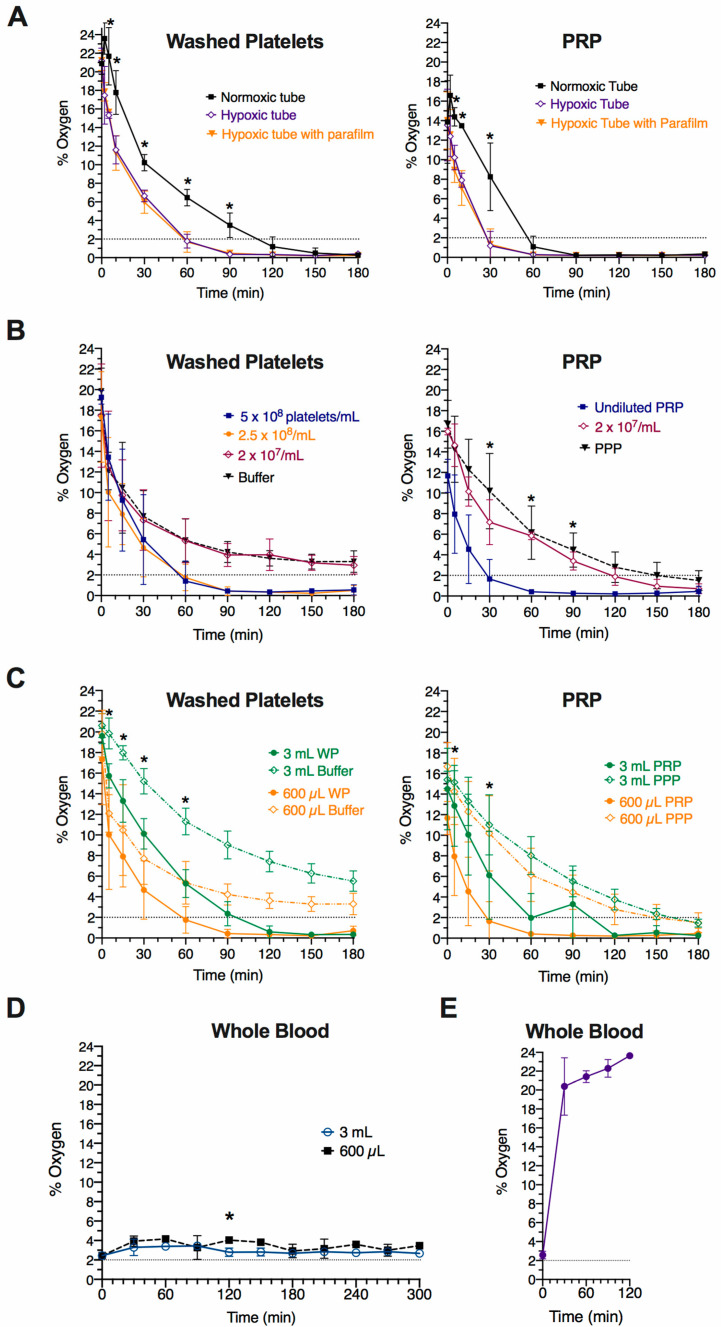
Effect of experimental conditions on oxygen level changes in platelet suspensions and whole blood incubated at 2% oxygen. Dissolved oxygen levels were measured using a fibre optic oxygen sensor in different samples as follows: (**A**) Comparison of type of container. A total of 600 µL of washed platelets (WP, 2.5 × 10^8^ platelets/mL) and undiluted platelet-rich plasma (PRP, 4–8 × 10^8^ platelets/mL) incubated at 2% oxygen in three different 1.5 mL tubes: partially closed tubes previously stored at atmospheric conditions (normoxic), partially closed tubes pre-equilibrated at 2% oxygen (hypoxic), and 2% oxygen pre-equilibrated tubes with parafilm covering the top (hypoxic with parafilm). Data shown as mean ± SD (*n* = 3). Statistical significance was calculated using two-way ANOVA repeated measures, followed by Tukey’s multiple comparison correction, with *p* ≤ 0.05 (*) shown for normoxic compared to hypoxic tubes. (**B**) Comparison of platelet concentrations. A total of 600 µL aliquots of WP and PRP at different concentrations, alongside modified Tyrode’s buffer and platelet-poor plasma (PPP), in hypoxia pre-equilibrated 1.5 mL tubes and incubated at 2% oxygen with the lids partially closed. Undiluted PRP ranged from 3.7 to 4.2 × 10^8^ platelets/mL. Data shown as mean ± SD (*n* = 3). Two-way ANOVA repeated measures, followed by Tukey’s multiple comparison correction, with *p* ≤ 0.05 (*) shown for WP/PRP highest platelet concentration compared to the rest. (**C**) Comparison of different volumes. WP (2.5 × 10^8^/mL), undiluted PRP (3.7–4.2 × 10^8^ platelets/mL), modified Tyrode’s buffer and PPP aliquoted in 3 mL and 600 µL using pre-equilibrated 15 mL and 1.5 mL tubes, respectively, and incubated at 2% oxygen with the lids partially closed. Data shown as mean ± SD (*n* = 3) for all data except 3 mL PPP (*n* = 2). Two-way ANOVA repeated measures, followed by Tukey’s multiple comparison correction, with *p* ≤ 0.05 (*) shown for 600 µL WP/PRP compared to 3 mL WP/PRP. (**D**) Whole blood incubated at 2% oxygen. Whole blood drawn in sodium citrate and immediately exposed to 2% oxygen and aliquoted in two different volumes using 2% oxygen pre-equilibrated plasticware: a total of 600 µL placed into a 1.5 mL tube kept still with the lid partially closed, and 3 mL placed into a well (35 mm diameter) of a 6 well culture dish with the lid on and kept gently shaken. Data shown as mean ± SD (*n* = 3). Two-way ANOVA repeated measures, followed by Sidak’s multiple comparison correction (* *p* ≤ 0.05). (**E**) Whole blood incubated at atmospheric conditions. A total of 1.4 mL of sodium citrated whole blood immediately placed into a 15 mL tube with the lid closed and rotated on a horizontal roller at 20 rpm in atmospheric conditions. Data shown as mean ± SD (*n* = 3). For all the above measurements, 2% and 21% oxygen pre-equilibrated distilled water was used as an internal control and read 2% and 21%, respectively, throughout the experiments.

As seen in Figure 1A, all three conditions reached 2% oxygen, but the platelet suspensions within pre-equilibrated tubes were the fastest, at 60 min for WP and 30 min for PRP. These times were approximately 50 min and 30 min faster than in non-equilibrated tubes, respectively. Notably, the dissolved oxygen levels in both the WP and PRP samples dropped below 2% oxygen, reaching values close to 0% at 90 and 60 min, respectively. Near-anoxic conditions can also take place in static in vitro cell cultures (typically incubated at 18.6% oxygen) and occur when the oxygen consumption rate of cells exceeds the diffusion rate of oxygen through the medium [18,19]. The faster de-oxygenation rate in PRP compared to WP might be due to the presence of proteins and other compounds in plasma (e.g., expected to reduce the total capacity for dissolved oxygen [19]) that are absent in the modified Tyrode’s buffer used to resuspend WP. However, other potential factors, such as increased platelet metabolism (and thus oxygen consumption) in PRP should also be considered.

In conclusion, here we found that both pre-equilibrating the tubes to reduced oxygen and the presence of plasma shorten the time for platelet sample de-oxygenation. Therefore, in subsequent experiments, we used tubes that had been exposed to 2% oxygen overnight prior to use. Importantly, our results also show that the true microenvironment to which platelets are exposed can be different from the one set up in the hypoxia chamber.

### 2.2. Effect of Platelet Concentration and Volume on Oxygen Level Changes in Platelet Suspensions Expose to 2% Oxygen

Platelet samples are usually prepared at different platelet concentrations and volumes depending on the assay to be performed. This includes physiological concentrations (for light-transmission aggregometry, flow cytometry, lysate collection for proteomic studies, etc.) but also sub-physiological platelet counts (for platelet spreading and flow cytometry), while volumes can vary typically from 50 µL for flow cytometry to 1 mL or more for cell lysates. Thus, we analysed whether cell concentration and volume might affect the de-oxygenation rate of platelet suspensions exposed to low oxygen.

Different concentrations of WP (5 × 10^8^, 2.5 × 10^8^ and 2 × 10^7^ platelets/mL) and PRP (undiluted ranging from 3.7 to 4.2 × 10^8^ platelets/mL, and 2 × 10^7^ platelets/mL) relevant to platelet testing were prepared, and the oxygen concentration (“Time 0”) was measured in these samples alongside the WP buffer and platelet-poor plasma (PPP). Once placed inside the 2% oxygen chamber, 600 µL aliquots of the samples were immediately prepared in 1.5 mL tubes that were kept partially closed during the incubation, except for when the oxygen concentrations were read (Figure 1B). For both WP and PRP, a trend was found in which higher platelet concentrations showed quicker de-oxygenation rates, which was statistically significant for PRP. WP at 2 × 10^7^ platelets/mL and cell-free modified Tyrode’s buffer did not reach 2% dissolved oxygen during the 180 min monitored, while PRP at 2 × 10^7^ platelets/mL and PPP reached 2% oxygen around 90 min and 120 min later than undiluted PRP, respectively (Figure 1B). In reference to the sample volume, WP at 2.5 × 10^8^ platelets/mL and undiluted PRP, alongside cell-free modified Tyrode’s buffer and PPP, were placed inside 2% oxygen pre-equilibrated tubes of two different sizes, 1.5 mL tubes (600 µL aliquots) and 15 mL tubes (3 mL aliquots) that were kept partially closed except when the oxygen measurements were taken. A trend of faster de-oxygenation in smaller volumes was found both for WP and PRP (Figure 1C). Regardless of volume, the WP and PRP samples dropped below 2% oxygen, as seen above (Figure 1A–C).

Altogether, our results show that both platelet concentration and volume affect the rate of de-oxygenation in platelet suspensions exposed to low oxygen levels, which can be explained by the slow oxygen diffusion through aqueous media combined to cell oxygen demand [19]. Compared to platelet suspensions, cell-free modified Tyrode’s buffer and PPP had slower de-oxygenation rates. In conclusion, care should be taken if preparing platelet samples and buffers at different conditions when, for example, performing different assays within a study, as these might not produce results that can be compared.

### 2.3. Changes in Oxygen Levels on Whole Blood Samples Exposed to 2% Oxygen

Some platelet function tests are performed in whole blood (e.g., impedance aggregometry, thrombus formation under flow, and flow cytometry). Considering the role of red blood cells (RBCs) in oxygen transport and delivery, we aimed to measure de-oxygenation rates in whole blood samples exposed to 2% oxygen. Blood obtained by venepuncture in sodium citrate anticoagulant was measured for oxygen concentration (Time “0”) and immediately placed in the hypoxia chamber and aliquoted in two different 2% oxygen pre-equilibrated containers: 1.5 mL tubes (600 µL aliquots) kept still with the lid partially closed as previously performed for PRP/WP, and 6 well culture plates (3 mL blood aliquot per well) with the lid on that were gently shaken using a VarioMag Monoshaker (Camlab, Cambridge, UK). Regardless of volume, container and shaking conditions, oxygen levels in whole blood remained close over time, fluctuating from 2.5–4.5% oxygen throughout the three hours that were monitored (Figure 1D). We also measured changes in oxygen levels when whole blood was kept at atmospheric conditions after venepuncture. To this end, a total of 1.5 mL of sodium citrated blood was placed into a 15 mL tube and incubated on a roller with gentle mixing at 30 rpm. The dissolved oxygen concentration was read every half hour for three hours; it reached 21% within the first 30 min (Figure 1E).

Therefore, when exposing whole blood to 2% oxygen, platelets do not experience oxygen concentrations below 2% for at least three hours, as opposed to what occurs in PRP and WP.

### 2.4. Changes in Oxygen Levels in Platelet Suspensions Kept at Atmospheric Conditions

After detecting that the WP and PRP samples, but not the whole blood, almost reached anoxia when exposed to 2% oxygen, we aimed to investigate whether de-oxygenation also takes place in platelet suspensions prepared and kept static at atmospheric 21% oxygen, as is routinely the case in human platelet studies.

**Figure 2:**
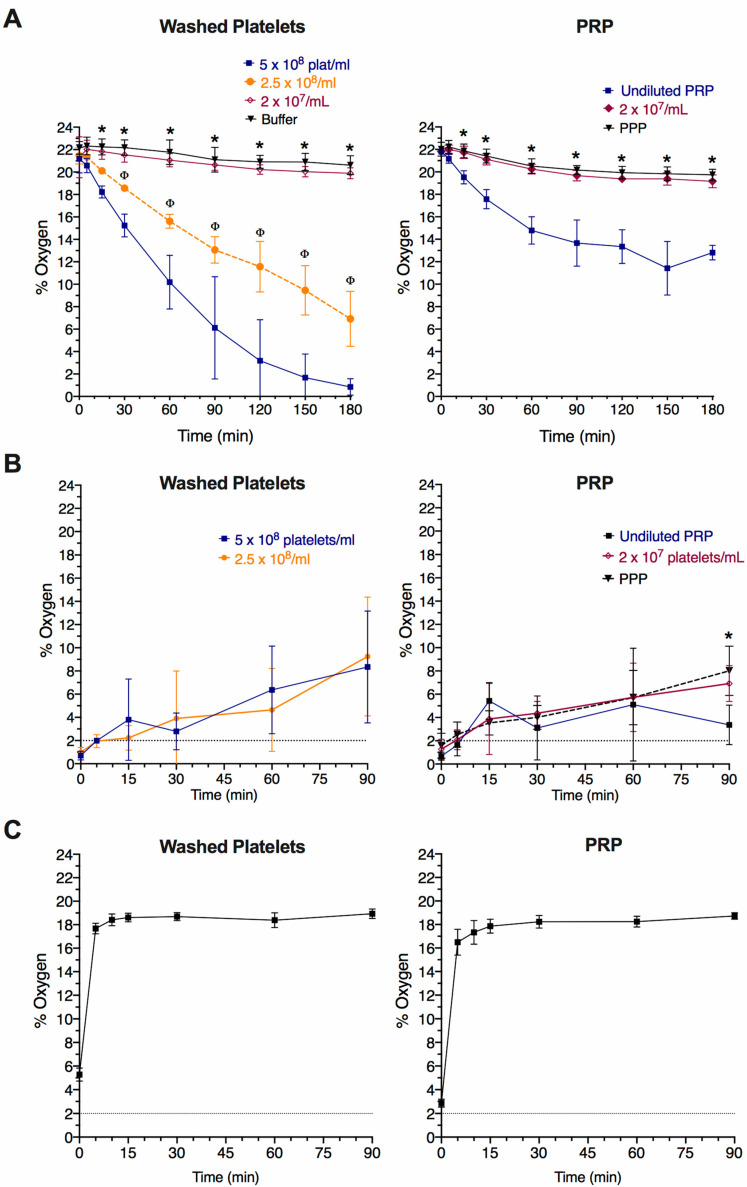
Effect of experimental conditions on oxygen level changes in platelet suspensions incubated at 21% oxygen. Dissolved oxygen levels were measured using a fibre optic oxygen sensor in different samples as follows: (**A**) Platelet suspensions kept at 21% oxygen. WP and PRP aliquots of 600 µL at different platelet concentrations, alongside modified Tyrode’s buffer and PPP, in 1.5 mL tubes left still with the lids closed, only being opened when dissolved oxygen measurements were taken. Undiluted PRP ranged from 2 to 3.5 × 10^8^ platelets/mL. Data shown as mean ± SD (*n* = 4 for WP and *n* = 3 for PRP). Statistical significance was calculated using two-way ANOVA repeated measures, followed by Tukey’s multiple comparison correction, with *p* ≤ 0.05 shown for WP/PRP highest platelet concentration compared to 2 × 10^7^ platelets/mL (*) and 2.5 × 10^8^ platelets/mL (Φ). (**B**) Pre-incubated platelet suspensions (2% oxygen) in static conditions. 600 µL WP and PRP samples at different platelet concentrations in 1.5 mL tubes that were incubated at 2% oxygen for three hours before being removed from the hypoxia chamber and left still at atmospheric 21% oxygen. Tubes were left with their lids partially closed, only being opened when dissolved oxygen measurements were taken. Undiluted PRP ranged from 3.8 to 6.4 × 10^8^ platelets/mL. Data shown as mean ± SD (*n* = 4). Two-way ANOVA repeated measures, followed by Sidak’s (for WP) or Tukey’s (for PRP) multiple comparison correction, with *p* ≤ 0.05 considered significant. No statistically significant differences were detected. (**C**) Pre-incubated platelet suspensions (2% oxygen) in stirring conditions. The re-oxygenation rate of WP (2.5 × 10^8^ platelets/mL) and PRP (undiluted, 2 to 3.5 × 10^8^ platelets/mL) pre-incubated at 2% oxygen for two hours was measured. When 600 µL samples were taken out from the chamber, 250 µL aliquots were transferred to an open cuvette, and oxygen levels were recorded immediately. Platelets were then stirred using a light transmission aggregometer and oxygen levels were read at different time points for 90 min. Data shown as mean ± SD (*n* = 3).

Freshly prepared WP and PRP suspensions were kept in a horizontal roller with a gentle rotation until the start of the assay (“Time 0”), in which 600 µL of WP and PRP were aliquoted in 1.5 mL polypropylene microcentrifuge tubes and incubated at 37 °C with the lid closed. As seen in Figure 2A, a platelet concentration-dependent decrease in dissolved oxygen concentration was observed in WP, with samples at 5 × 10^8^ platelets/mL reaching 1% oxygen or below after 3 h. By contrast, a milder decrease was detected in the undiluted PRP with dissolved oxygen concentration, which dropped to 12–14% over the same period.

These results indicate that care should also be taken when preparing platelet samples intended to be kept at 21% oxygen, especially if they are to be used for comparison to samples exposed to low oxygen in hypoxia chambers.

### 2.5. Re-Oxygenation of Hypoxia-Treated Platelet Suspensions upon Exposure to Atmospheric Conditions

Performing human platelet research in hypoxic conditions presents technical challenges as some pieces of equipment, like light-transmission aggregometers, cannot easily be fitted inside most cell culture hypoxia workstations. However, it is unknown how oxygen concentrations change in hypoxia-exposed platelet suspensions when they are subsequently exposed to atmospheric conditions for testing. Thus, we analysed the re-oxygenation rate of hypoxic platelet samples in still and stirring conditions in an atmospheric environment.

Samples of WP (2.5 × 10^8^ platelets/mL) and undiluted PRP, each of 600 µL, were removed from the hypoxia chamber after 2 h of exposure to 2% oxygen and allowed to rest at atmospheric conditions at 37 °C, keeping the tubes partially closed between oxygen readings. As shown in Figure 2B, although the oxygen concentration increased over time, none of the samples reached 21% oxygen within 90 min. To mimic the impact of preparation for light-transmission aggregometry studies, 600 µL-aliquots of WP (2.5 × 10^8^ platelets/mL) and undiluted PRP were removed from the hypoxic chamber, 250 µL of which was transferred into open cuvettes (P/N 312, Chrono-log Corporation, Pennsylvania, USA) and stirred at 1200 rpm at 37 °C in a CHRONO-LOG^®^ Model 700 aggregometer (Chrono-log Corporation). Dissolved oxygen was measured in the cuvettes before and after 5 min of stirring (e.g., the time that an average aggregation reaction would take place) and intervals up to 90 min. Both the WP and PRP samples increased to around 16% oxygen levels after 5 min of stirring (Figure 2C). These observations should be taken into consideration when designing experiments; hypoxic platelets are rapidly exposed to hyperoxic conditions in a stirred system, which may impact interpretation of data.

### 2.6. Platelet Spreading on Fibrinogen at Atmospheric and 2% Oxygen

Our previous data indicate that experimental conditions affect the oxygen levels that platelets experience at the cellular level. To test whether experimental conditions could also affect the results of platelet function tests, we performed platelet spreading assays that can be done inside the hypoxia chamber. Platelet spreading is important in maintaining vascular integrity and thrombus formation, but very little is known about how it is affected by hypoxia [16].

The platelet spreading on fibrinogen was performed in parallel in two conditions: (1) at 2% oxygen using 2% oxygen pre-equilibrated WPs, and (2) at atmospheric conditions with WPs kept at 21% oxygen. To prepare the hypoxic WP, 600 µL at 2.5 × 10^8^ platelets/mL were kept at 2% oxygen for 90 min before being diluted to 2 × 10^7^ platelets/mL in 2% oxygen pre-equilibrated buffer. For each oxygen environment (2% and 21%), the WP were allowed to spread for 1 h at 37 °C on two different 24 well plates: one hypoxia pre-equilibrated plate containing coverslips coated with fibrinogen at 2% oxygen, and one atmospheric-equilibrated plate containing coverslips coated with fibrinogen at 21% oxygen.

**Figure 3:**
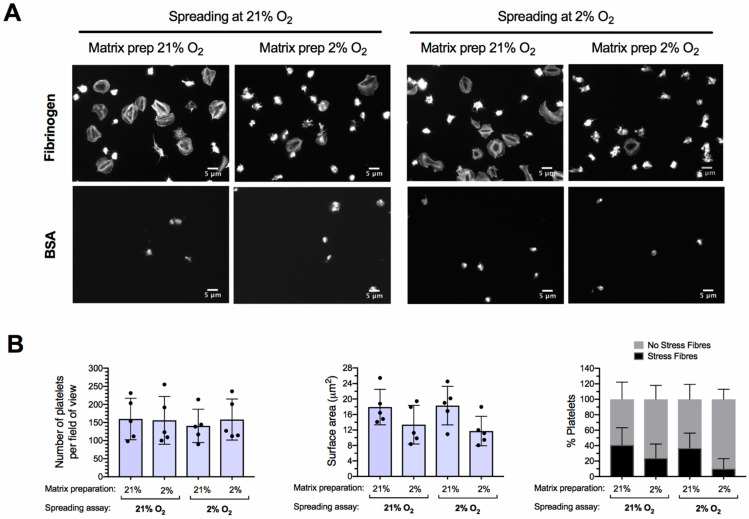
Effect of experimental conditions on platelet spreading on fibrinogen at 2% oxygen. Washed platelets at 2.5 × 10^8^ platelets/mL were incubated at 2% oxygen or atmospheric conditions for 90 min before being diluted to 2 × 10^7^ platelets/mL with modified Tyrode’s buffer pre-equilibrated to the same oxygen percentage and allowed to spread for one hour on immobilised fibrinogen or BSA. In each case, coverslips freshly coated with fibrinogen or BSA at 2% and 21% oxygen using overnight oxygen pre-equilibrated culture plates were tested. After the incubation time, unbound platelets were washed away and samples were fixed with 4% paraformaldehyde. At this point, samples exposed at 2% oxygen were removed from the hypoxia chamber for permeabilisation, FITC-phalloidin staining and visualisation by fluorescence microscopy. (**A**) Representative images of one out of 5 replicates are shown. (**B**) Mean ± SD of number of adhered platelets, surface area, and percentage of platelets with stress fibres (*n* = 5). Statistical significance was calculated using two-way ANOVA, followed by Tukey’s multiple comparison correction, with *p* ≤ 0.05 considered significant. No statistically significant differences were detected.

As shown in Figure 3, no changes in the number of platelets were observed among the four conditions. However, although not statistically significant, a trend of decreased platelet surface area and percentage of platelets with stress fibres, suggesting impaired activation, was detected in the assays performed with 2% oxygen pre-equilibrated plates, regardless of the oxygen percentage in the external environment where the platelet incubation took place. This suggests that platelet spreading on fibrinogen is compromised by the oxygen percentage at which the fibrinogen matrix and containers are prepared.

## 3. Discussion

This study showed that the rate at which dissolved oxygen changes in human platelet suspensions exposed to low oxygen concentrations depends on several experimental factors, including the type of container, platelet concentration, sample volume, presence of plasma and, potentially, the presence of RBCs. Importantly, we detected nearly anoxic levels in some platelet suspensions exposed to both 2% and 21% oxygen, demonstrating that the oxygen concentrations that platelets experience at the cellular level in vitro do not necessarily match the prevailing atmospheric conditions. This finding is relevant not only to hypoxia-focused research, but also to a larger number of human platelet investigations, as platelet suspensions are routinely prepared and kept static at 21% oxygen. As reported for nucleated cell cultures, the dissolved oxygen observations presented here can be explained by the solubility properties of molecular oxygen, and the fact that its slow rate of diffusion through aqueous media can be exceeded by the oxygen consumption rate of cells [19]. Thus, our study revealed similar limitations to those found in cell cultures even when using platelet suspensions for short times (from a few minutes to a few hours). The results shown here should be taken into consideration when designing future studies. This should include the preparation of platelet samples but also buffers and other relevant solutions, as oxygen equilibration in cell-free medium is known to be a slow process, requiring significant time periods even when using perforated lids if containers are kept still (without shaking) [20,21]. Factors such as temperature, ionic strength, and protein concentration should also be considered as they affect oxygen solubility [19]. Some strategies that are used to help circumvent the problem of oxygen diffusion in cell cultures could also be adapted for platelet suspensions; for example, keeping the samples in gentle shaking or using culture plates with oxygen permeable plastic membranes at the bottom of the wells [19].

Despite technical challenges, incubating and testing isolated human platelets in hypoxia chambers is a useful strategy that allows the characterisation of the direct and quick effect of specific oxygen levels on platelets. Although some platelet alterations might be the result of several days of exposure to hypoxia, Cameron et al. [15] reported changes in the expression of platelet proteins after a few hours of exposure to 5% oxygen. Moreover, Chaurasia et al. [17] found increasing levels of HIF-2α upon platelet exposure to 1% oxygen from 30 to 120 min of testing. They also detected an enhanced expression of HIF-2α in platelets kept at atmospheric conditions and stimulated with physiological agonists, namely thrombin, ADP and collagen. Although published studies have used different sample preparations and oxygen percentages [15,16,17], the actual oxygen concentrations in the platelet suspensions at the time when the results were collected were not reported. Based on our study, it is plausible that some of these samples reached near-anoxia.

Because of the re-oxygenation of low-oxygen-exposed platelets once returned to atmospheric conditions, especially when stirring or when working with whole blood, it is advisable to perform functional tests within the hypoxia workstation. One limitation of removing samples from hypoxic conditions is that potentially quick and reversible effects of low oxygen levels on platelet function and intracellular signalling might be missed. This limitation also applies to studies performed with samples obtained from hypoxia-related donors and healthy volunteers that are analysed at atmospheric conditions. Indeed, much of our knowledge of human platelet function is derived from ex vivo studies, where platelets are exposed to levels of atmospheric oxygen that do not match the prevailing conditions to which platelets are normally exposed in vivo. However, the importance and challenges of controlling experimental conditions for functional tests in low-oxygen environments should not be underappreciated. This can be seen in our platelet spreading assays, in which the use of hypoxic fibrinogen-coated culture plates showed a trend for decreased platelet surface area and stress fibres, even when the actual assay was performed at atmospheric conditions.

Notably, in this study, we observed that the oxygen levels in whole blood exposed to 2% oxygen did not drop below this oxygen concentration, unlike in WP and PRP, suggesting that RBCs or other factors in whole blood might be able to protect platelets from low oxygen stress. RBCs act as sensors of oxygen and, in response to hypoxia, they release compounds such as ATP that can affect platelet function [22,23]. Moreover, platelet–RBC interactions are known to contribute to thrombus formation [24]. Therefore, it is important to conduct future investigations on platelet function at sub-atmospheric oxygen conditions in the presence of RBCs.

In conclusion, our study shows the importance of carefully choosing, testing and reporting experimental conditions for future investigations on the role of hypoxia in platelet function. This information is key for the interpretation of results and comparison across studies.

## 4. Materials and Methods

### 4.1. Reagents

All the reagents were from Merck (Sigma-Aldrich, Darmstadt, Germany) unless otherwise indicated.

### 4.2. Human Samples and Ethical Considerations

This study was performed in accordance with Hull York Medical School Ethics Committe (reference number 1501). Informed consent was obtained from all the participants, consisting of healthy volunteers over 18 years of age who had not taken anti-platelet agents for the previous ten days. Blood was collected by venepuncture in syringes containing sodium citrate or ACD to obtain PRP or WP, respectively.

### 4.3. Platelet Preparation and Hypoxia Incubation

The PRP was obtained through the centrifugation of sodium citrated blood at 190× *g* for 15 min, followed by the transfer of the upper phase to a fresh tube. Platelet-poor plasma (PPP) was prepared from an aliquot of PRP that was centrifuged at 1700× *g* for 5 min; the platelet pellet was discarded.

For the WP preparation, whole blood in ACD was first used to obtain the PRP, as above. Citric acid (6 mM) was then added to the PRP before being centrifuged at 800× *g* for 12 min. The pellet was re-suspended in a wash buffer (36 mM citric acid, 10 mM EDTA, 5 mM D-glucose, 5 mM KCl, 90 mM NaCl) and centrifuged again. The pellets were then resuspended in a modified Tyrode’s buffer (20 mM HEPES, 134 mM NaCl, 2 mM KCl, 0.34 mM Na_2_HPO_4_, 12 mM NaHCO_3_, 1 mM MgCl_2_, 5 mM glucose) at different platelet concentrations (2 × 10^7^/mL to 5 × 10^8^/mL) depending on the assay (see the Results for details).

When required, the whole blood, PRP and WPs were exposed to 2% oxygen (5% CO_2_) in a Whitley H35 Hypoxystation (Don Whitley Scientific, Bingley, UK) at 37 °C in a humidified atmosphere. The platelet preparations at atmospheric conditions were also incubated at 37 °C. 

### 4.4. Measurement of Dissolved Oxygen in Cell Suspensions

Dissolved oxygen in buffers, platelet suspensions and whole blood was measured using an OXY-4 ST oxygen meter attached to a Needle-Type Oxygen Microsensor NTH-PSt7 (PreSens, Regensburg, Germany). The oxygen levels of distilled water kept in atmospheric conditions (21% oxygen) and 2% oxygen conditions were measured regularly as controls.

### 4.5. Platelet Spreading Assay

Glass coverslips were placed inside the wells of 24 well plates and coated with 100 μg/mL human fibrinogen (depleted from plasminogen, von Willebrand Factor and fibronectin; Enzyme Research Laboratories, Swansea, UK) for 1 h at room temperature. The coverslips were then washed with PBS, blocked with 0.5% heat-denatured fatty acid free BSA for 1 h, and washed with PBS. Some coverslips were treated with BSA only and used as negative controls for platelet spreading. The coating of the coverslips was performed in two ways: at atmospheric conditions and at 2% oxygen. For the latter, the coverslips, 24 well plates and PBS were kept at 2% oxygen for at least 15 h before use. Some of the plates coated at 21% and 2% oxygen were swapped from atmospheric to hypoxic conditions and vice versa and used immediately for platelet spreading (see the Results for more information).

The spreading assays were performed at 2% and 21% oxygen conditions in parallel. For the hypoxic assays, 600 µL of washed platelets at a concentration of 2.5 × 10^8^ platelets/mL were first incubated at 2% oxygen for 90 min in 2% oxygen pre-equilibrated 1.5 mL microcentrifuge tubes. The platelet suspension was then diluted to a concentration of 2 × 10^7^/mL using 2% oxygen pre-equilibrated modified Tyrode’s buffer, and immediately incubated for 1 h with immobilised fibrinogen on glass coverslips. For spreading at 21% oxygen, the same protocol was followed, except the platelets were prepared and kept at 37 °C at atmospheric conditions. In both cases, unbound platelets were removed by gently washing with PBS. The coverslips were fixed with 4% paraformaldehyde for 10 min. At this point, the plates were removed from the hypoxic chamber and the rest of the protocol took place at atmospheric conditions. The samples were permeabilised with 0.3% Triton X-100 for 5 min, stained with FITC-phalloidin (ThermoFisher Scientific, Waltham, MA, USA) for 20 min and mounted onto glass slides using Invitrogen ProLongTM Diamond Antifade Mountant (ThermoFisher Scientific).

The coverslips were processed blinded and imaged at random using a Zeiss Axio Observer fluorescence microscope equipped with ApoTome structured illumination, a 63x oil immersion objective and an AxioCam 506 camera (Zeiss, UK). The images were analysed blinded using ImageJ (NIH, USA) and Zen Pro software (Zeiss UK).

### 4.6. Statistical Analysis

GraphPad Prism was used to perform the statistical analysis, including D’Agostino and Pearson and Shapiro–Wilk tests for normality. The data are presented as the mean ± SD. See the figure legends for further information.

## Data Availability

The data presented in this study are available on request from the corresponding author.
